# Surgery and Prophylaxis with Susoctocog-Alfa in Acquired Hemophilia: Case Series and Literature Review

**DOI:** 10.3390/jcm12144590

**Published:** 2023-07-10

**Authors:** Carola Sella, Marco Bardetta, Federica Valeri, Cristina Dainese, Alessandra Valpreda, Massimo Massaia, Daniele Grimaldi, Annamaria Porreca, Benedetto Bruno, Alessandra Borchiellini

**Affiliations:** 1 Regional Centre for Hemorrhagic and Thrombotic Diseases, AOU Città della Salute e della Scienza, 10126 Turin, Italy; mbardetta@cittadellasalute.to.it (M.B.); fvaleri@cittadellasalute.to.it (F.V.); cdainese@cittadellasalute.to.it (C.D.); aborchiellini@cittadellasalute.to.it (A.B.); 2 Division of Hematology, AOU Città della Salute e della Scienza, University of Turin, 10126 Turin, Italy; benedetto.bruno@unito.it; 3 Department of Molecular Biotechnology and Health Sciences, University of Turin, 10124 Turin, Italy; 4 Central Laboratory Baldi and Riberi, AOU Città della Salute e della Scienza, University of Turin, 10124 Turin, Italy; avalpreda@cittadellasalute.to.it; 5 Hematology Unit, Santa Croce e Carle Hospital, 12100 Cuneo, Italy; massimo.massaia@unito.it (M.M.); daniele.grimaldi@unito.it (D.G.); 6 Department of Medical, Oral and Biotechnologies Science, University of Chieti-Pescara, 66100 Chieti, Italy; a.porreca@hnpgroup.eu

**Keywords:** acquired hemophilia A, recombinant porcine FVIII, surgery, prophylaxis

## Abstract

Background: Acquired hemophilia A (AHA) is a rare bleeding disease due to autoantibodies directed against clotting factor VIII (FVIII). Treatment of AHA consists of inhibitor eradication with immunosuppressive therapy (IST) and prompt control of bleeding obtained with bypassing agents or recombinant porcine FVIII (rpFVIII). The latter has recently been licensed for management of acute bleeding in AHA. Unlike treatment with bypassing agents, rpFVIII can be monitored to provide a successful hemostatic effect and avoid overtreatment. Correlation between rpFVIII inhibitor titers and efficacy of rpFVIII treatment remains a matter of debate. Methods: We report three cases of AHA in which rpFVIII was successfully used with an unconventional schedule despite the presence of medium–high titers of the rpFVIII. The modified Nijmegen–Bethesda inhibitor assay (NBA) was used to dose porcine FVIII inhibitors. Result: The presence of rpFVIII inhibitors prior to the exposition to susoctocog-alfa, that may suggest a cross-reactivity with human FVIII inhibitors, did not affect hemostasis. Conclusion: In our experience, rpFVIII demonstrates safety and efficacy in the presence of rpFVIII inhibitors and using an unconventional schedule in both the perioperative and outpatient settings. Laboratory measurement of inhibitors against rpFVIII during treatment is described for the first time.

## 1. Introduction

Acquired hemophilia A (AHA) is a rare bleeding disorder caused by autoantibodies which interfere with the activity of clotting factor VIII (FVIII). It affects males and females with previously normal hemostasis [1]. AHA is often caused by underlying clinical conditions, such as autoimmune disorders, malignancy, cutaneous diseases (pemphigus and psoriasis), drugs, and pregnancy [2]. In addition, coronavirus 2-associated disease 2019 (COVID-19) and relative vaccinations have recently been described as trigger conditions [3,4]. The incidence of AHA has been estimated at about 1.5 cases per million/year, with prevalence in people over 65 years old. Currently, its mortality is above 3–15% [5,6,7]. The bleeding site is more frequently subcutaneous followed by gastrointestinal, muscular, and genitourinary, while joint bleeding is less common as compared to congenital hemophilia [5,6].

The management of AHA should be focused on prompt control of bleeding, inhibitor eradication, and, when possible, treatment of the underlying disease [1]. Hemostatic treatments include recombinant activated factor VII (rFVIIa), activated prothrombin complex concentrate (aPCC), both known as bypassing agents, and susoctocog-alfa, a recombinant porcine FVIII (rpFVIII, Obizur^®^) [1,8]. The latter has recently been licensed for management of acute bleeding in AHA [9] based on a prospective study in adult patients with major bleedings [10]. rpFVIII advantages include the possibility of monitoring response with standard coagulation laboratory assays and minimizing thrombotic risk [11,12,13,14,15]. Thromboembolic risk is, indeed, increased in patients treated with bypassing agents, particularly the elderly and those with underlying pro thrombotic conditions (i.e., previous thrombosis, immobilization, malignancy, indwelling catheters, surgery, and pregnancy/post-partum) [7,16,17].

In pivotal studies in patients with congenital hemophilia A with inhibitors and in patients with acquired hemophilia, the loading dose utilized was 200 IU/kg, followed by median dose of 100 IU/kg [10]. Nevertheless, data on real-life use of susoctocog-alfa, recently collected in small-case series, confirmed safety and efficacy of lower starting doses (100–120 IU/kg) [18,19,20].

The role of dosing rpFVIII inhibitors prior to the administration of Obizur^®^ in the clinical management of AHA remains controversial. Several authors suggest determining anti-rpFVIII cross-reactivity before starting treatment and monitoring any development of rpFVIII inhibitors during therapy [1,21,22,23,24]. In contrast, other studies show that rpFVIII is effective even in the presence of anti-rpFVIII [10,20,25,26,27]. Due to the limited number of randomized studies on AHA management because of the rarity of the disease, other open questions remain, especially on the safety and efficacy of rpFVIII treatment in certain clinical conditions such as surgical interventions, and perioperative and ambulatory settings.

Here, we report our real-life experience on three cases of AHA in unconventional settings, also having rpFVIII inhibitors before treatment. Furthermore, we review similar case reports from literature aiming to compare rpFVIII efficacy.

## 2. Materials and Methods

On the first day of treatment, rpFVIII levels were measured pre-infusion and at 1, 3, and 6 h after administration in all patients. Regular monitoring was performed on the following days by measuring baseline and immediate post-infusion levels. rpFVIII levels were quantified by one-stage assay (OSA) using human plasma for the calibration curve, as per the manufacturer’s instructions. HemosIL calibration plasma (Werfen, Bedford, MA, USA) with SynthasIL, with silica as activator, were used. Since no specific-quality control plasma for rpFVIII activity is available, rpFVIII standard (Takeda Company, Bannockburn, IL, USA) diluted in deficient plasma up to the concentration of 1 UI/mL was tested and used as control.

In order to investigate the presence of cross-reacting rpFVIII inhibitors, anti-rpFVIII titration was performed with a validated modified Nijmegen–Bethesda anti-human FVIII assay where standard rpFVIII (Takeda) was used as substrate. Commercial plasmas with known anti-human factor inhibitors (Technoclone GmbH, Vienna, Austria) were used as controls.

## 3. Case Reports and Results

We report three cases of AHA diagnosed and treated with rpFVIII (Obizur^®^—Takeda Pharmaceutical Co., Tokyo, Japan) between January and May 2021 in two Italian tertiary centers where rpFVIII was used despite the presence of rpFVIII inhibitors. Patient characteristics, treatment schedule, and safety are summarized in Table 1, Table 2 and Table 3.

### 3.1. Case Reports

#### 3.1.1. Case 1

A 70-year-old woman with a history of myasthenia gravis, Hashimoto thyroiditis, and glaucoma was hospitalized for right-upper-limb hematoma. Coagulation tests showed prolongation of activated partial thromboplastin clotting-time ratio (aPTTr) not corrected by mixing test, FVIII 0.9% and FVIII inhibitors titer 76 UB/mL. Diagnosis of AHA was made. IST with steroids (followed by addition of cyclophosphamide because of lack of response) was promptly administered. No hemostatic therapy was initially needed given stable hemoglobin levels and absence of new bleeding episodes. During hospitalization, because of an acute onset of abdominal pain, a CT-scan was performed and revealed a diverticular bowel perforation. She underwent surgery on rFVIIa coverage without complications. On the fourth post-operative day, due to elevated rFVIIa dependency, rpFVIII was administered despite presence of a medium–high rpFVIII inhibitor titer (44 UB/mL). The initial schedule consisted of 200 U/kg every 12 h, reaching a FVIII recovery level of 125% 15 min after the first dose. Time between administrations was then gradually prolonged (every 24–36–48 h) to maintain FVIII level up to 60%. When dose de-escalation was started, human FVIII inhibitors titer was 6 UB/mL. She had no AHA relapse, adverse events, or hemorrhagic complications. rpFVIII inhibitors titer kept decreasing during treatment and 24 days from the first Obizur^®^ dose the titer was 2.0 UB/mL. The patient was discharged 50 days after with FVIII at 268% and FVIII inhibitors titer of 0 UB/mL. No relapse has occurred up to follow-up.

#### 3.1.2. Case 2

A 79-year-old man with a history of gastric carcinoma and polymyalgia rheumatica was referred to our center for hematuria that required blood transfusions after bladder biopsies, performed for urothelial neoplasia suspicion. He also presented with diffuse cutaneous hematomas and a prolonged aPTTr (2.57) not corrected by mix test. AHA diagnosis was made given a FVIII residual activity of 1.7% and a FVIII inhibitors titer of 23 UB/mL. Hemostatic treatment was promptly started with rFVIIa. For inadequate response and persistent hematuria, the bypassing agent was replaced by rpFVIII at a dose of 200 U/kg. rpFVIII inhibitor titer was 12 UB/mL and FVIII recovery 30 min after the first administration was 94%. FVIII trough level remained stabled around 10% that allowed bleeding control. When FVIII and human FVIII inhibitor titer were 32% and 2.1 UB/mL, respectively, he underwent ureteroscopy with biopsy and laser ablation of the ureter neoplastic lesion under rpFVIII coverage (at a dose of 100 U/kg) without complications. In the meantime, complete eradication of inhibitors was achieved after three lines of IST (steroids, cyclophosphamide, and Rituximab), given the persistence of inhibitors high titers. He was discharged with FVIII 153% and FVIII inhibitor titer of 0.66 UB/mL. At last follow-up, he remains in complete remission with an aPTTr of 0.96 and a FVIII of 154%.

#### 3.1.3. Case 3

A 31-year-old woman with unremarkable medical history was admitted to the emergency room for right-leg pain one month after COVID-19 infection and two months after childbirth. She was initially treated with a single intramuscular injection of NSAID in the gluteus. Five days later, she returned to emergency room for asthenia with anemia (hemoglobin 6 g/dL). A CT-scan showed right-lower-limb hematoma. Diagnosis of post-partum AHA was promptly made as laboratory tests revealed an aPTTr of 3.0 not corrected by mixing test, FVIII activity of 1% and FVIII inhibitors titer of 64 UB/mL. Therapy with rFVIIa and steroids was started with immediate response. She was discharged eight days later. However, five days later, she presented again with a new episode of bleeding in the ilio-psoas muscle which was initially treated with rFVIIa, then replaced with rpFVIII (loading dose of 200 U/kg followed by 100 U/kg). A medium–low anti-rpFVIII inhibitors titer (11.2 UB/mL) was found before Obizur^®^ was started. aPTTr normalization was, however, promptly obtained and, 30 min after the first infusion, FVIII plasmatic level reached 213%. Hemoglobin levels progressively increased and no other bleeding episodes were observed. After 13 days, the patient was discharged with FVIII inhibitors titer of 3.1 UB/mL. Susoctocog-alfa infusions were administered as outpatient at reduced doses (100 U/kg every other day) maintaining FVIII levels up to 30% given the large hematoma and high risk of rebleeding. Steroids were stopped six months later with no recurrence of AHA or adverse events. In this patient, rituximab was not administered, firstly, because she was breastfeeding, secondly, because post-partum AHA is frequently associated with favourable outcome, and, above all, because of the ongoing first COVID-19 pandemic wave.

### 3.2. Results

Here, we report our strategy using susoctocog-alfa in three patients with baseline cross-reacting anti-porcine FVIII antibodies, high thrombotic risk, and need of prophylactic anti-hemorrhagic treatment. The hemostatic effect was achieved in all patients and no adverse events were observed, including, most importantly, thrombosis. FVIII was monitored before and after rpFVIII administration and dose adjustment was applied as clinically indicated to avoid under- or overtreatment. Table 4 illustrates our treatment with Obizur^®^ in the periprocedural setting and two other reports. Zanon et al., describes efficient rpFVIII coverage (FVIII trough 50%) during cholecystectomy performed 25 days after AHA diagnosis and concomitant unstable angina [25]. Buczuma et al., describes AHA management diagnosed three years before ocular surgery for entropion (FVIII trough 80%) [28].

Furthermore, we described the outpatient use of rpFVIII as post-partum prophylaxis to prevent recurrent bleeding in a young woman. Only two cases have, so far, been reported using rpFVIII after discharge. Tarantino et al., reported on two patients, one of whom died and the other achieved complete remission [18] (Table 5).

**Figure 1:**
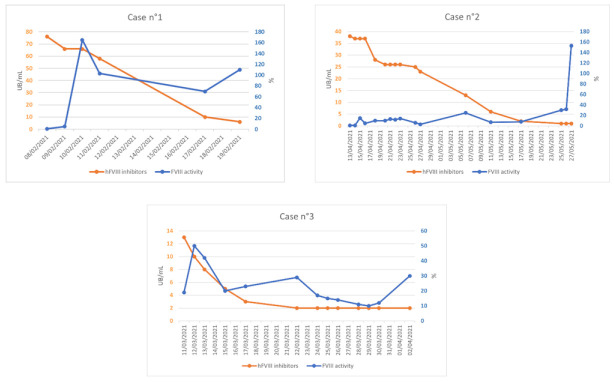
Factor VIII (FVIII) activity (%) and human factor VIII (hFVIII) inhibitors titer (UB/mL) during rpFVIII infusion.

Recovery rate was optimal using the initial standard dose despite the presence of baseline anti rpFVIII antibodies. Hemostatic efficacy was maintained allowing dose reduction with stable factor FVIII trough level at 60%, 10%, and 30%, respectively, for all three patients (Table 2). Given the optimal recovery, we were able to gradually reduce the infusion dose with a schedule similar to that used in congenital hemophilia, overall unusual in AHA (Figure 1).

## 4. Discussion

Even though AHA definitive treatment consists of autoantibodies eradication through immunosuppression, hemostatic therapy is necessary as front-line strategy for severe bleedings. Ideal hemostatic agent must be effective, safe, and, hopefully, monitorable. By-passing agents have displayed high efficacy, with control of bleeding in 90% of cases [16] for both rFVIIa and aPCC. However the main adverse event, though rare, is thrombosis, occurring in 3–7% of patients [16,17]. Furthermore, monitoring of bypassing agents is not available. rpFVIII was licensed in 2015 and real-life clinical experience is growing, in particular, of bleeding control [19,20,21,29], with a higher response when used as first-line therapy and on its safety in prothrombotic conditions given that no thromboembolic events have been reported.

However, since the number of patients treated is low (approximately 63 reported cases), the effect of cross-reacting antibodies on efficacy remains a matter of debate. Experience on the prophylactic use before and after surgery or in high-risk conditions of bleeding relapse is limited. Various dose regimens have been published, possibly due to different and complex dynamics in patients with inhibitors and/or to comorbidities, surgery, or inflammatory conditions [26].

In our experience, safe hemostasis was observed using susoctocog-alfa in the peri-procedure period even in the presence of baseline cross-reacting anti-rpFVIII antibodies. rpFVIII dose did not exceed 200 U/kg/d also in patients with high bleeding risk (Table 4). Thus, the possibility to dose rpFVIII allows to personalize hemostatic strategy depending on individual risk and recovery.

In AHA, the risk of bleeding recurrence persists until inhibitors are present [30]. To prevent this complication, Zanon et al., showed that using aPCC as prophylaxis until inhibitors are eradicated prevents bleeding complications. Similarly, we described the use of rpFVIII. In particular, Obizur^®^ was administered in a young woman in the post-partum period as outpatient to prevent bleeding relapse. Only two other cases described on rpFVIII administration after discharge have been reported. This schedule represents a favorable cost–benefit approach for patients with high risk of re-bleeding.

Another open question regards rpFVIII inhibitors and their clinical impact: in fact, data on in vivo correlation between inhibitor titers and hemostatic efficacy of rpFVIII are still lacking. Even though some authors recommend dosing rpFVIII inhibitors to raise the Obizur^®^ dose or avoid its use in case of high titers [1,22,23,24], in our patients, we obtained complete hemostasis, even in the presence of rpFVIII inhibitors at standard rpFVIII dose, in agreement with other reports [19,20].

Moreover, there is no standardized titration technique of anti-rpFVIII antibodies. Here, for the first time, we have described a laboratory method used to measure anti-rpFVIII antibodies during treatment in real-life, in agreement with published guidelines, using commercially available human FVIII inhibitor plasma [1,22,31,32].

## 5. Conclusions

Our experience supports efficacy and safety of susoctocog-alfa in the peri-surgical settings, even in the presence of rpFVIII inhibitors, without increasing the loading dose. Furthermore, total consumption is comparable to previous reports where rpFVIII inhibitors were not present and where different trough levels were required. Indeed, a further chance to reduce costs and improve quality of life of AHA patients is represented by an outpatient management. In this setting, an accurate FVIII monitoring is essential to avoid overtreatment and to prevent excessive costs. rpFVIII inhibitor detection and follow-up are also required. Here, we have reported for the first time methods of rpFVIII inhibitors titration in real-world practice that may be useful to further optimize laboratory methods. Nevertheless, there is the need to collect more data to better define the role of rpFVIII inhibitors in clinical practice and the profile of patients who may most benefit from outpatient treatment. Finally, a standardized method to dose rpFVIII inhibitors should be strongly sought.

## Figures and Tables

**Table 1:** Baseline patients’ characteristics.

	Case 1	Case 2	Case 3
Gender	Female	Male	Female
Age (years)	70	79	31
Comorbidities	Myasthenia gravis, Hashimoto Thyroiditis and Glaucoma	Gastric Carcinoma, Urothelial neoplasia, Polymyalgia rheumatica	Episode of major depression after son’s death
Thromboembolic risk factor	Surgery, age, prolonged immobilization	Surgery, neoplasia, age, prolonged immobilization	Post-partum
Bleeding type/site	Right-upper-limb Hematoma	Ilio-psoas hematoma, upper and lower limbs hematomas, hematuria	^1^ Right-lower-limb hematoma; ^2^ ilio-psoas hematomas
Surgery during AHA	Left hemicolectomy with terminal stomia	Tissue biopsy and laser ablation in the nearby region of neoplastic lesion	None
aPTT ratio	3.07	2.57	^1^ 3.26; ^2^ 1.8
FVIII at diagnosis (%)	0.9	1.7	^1^ 1; ^2^ 19
hFVIII inhibitors titer (UB/mL)	76	23	^1^ 64; ^2^ 11.2
rpFVIII inhibitors titer Pre-therapy (UB/mL)	44	12	11

AHA: Acquired hemophilia A; aPTT: activated partial thromboplastin time; hFVIII: human factor VIII of coagulation; rpFVIII: recombinant porcine factor VIII; UB: Unit Bethesda. ^1^ First hospitalization; ^2^ second hospitalization.

**Table 2:** Treatment features.

	Case 1	Case 2	Case 3
Lines of IST	2	3	1
Type of IST	Steroid, cyclophosphamide	Steroid, cyclophosphamide and rituximab	Steroid
First hemostatic therapy with rFVIIa dose (mcg/kg)	90	90	90
Days on rFVIIa treatment	4	5	^1^ 7 and ^2^ 3
Second hemostatic therapy	rpFVIII	rpFVIII	rpFVIII
rpFVIII loading dose (U/kg)	200	200	200
Recovery FVIII level within 1 h (%)	125	94	213
rpFVIII following dose (U/kg)	200	100	100
FVIII trough level (%)	60	10	30
Treatment schedule	Prolongation of administration interval (every 12–36–48 h) until ending	Full dose and half dose on alternate days and then half dose until ending	Half dose starting from 36 h after first dose, then half dose every other day
Days on rpFVIII treatment	11	44	22
Total treatment dose (U)	125.000	155.000	55.000

IST: immunosuppressive therapy; rFVIIa: recombinant factor VII activated; rpFVIII: recombinant porcine factor VIII; FVIII: factor VIII of coagulation. (^1^ First hospitalization; ^2^ second hospitalization).

**Table 3:** Safety and efficacy.

	Case 1	Case 2	Case 3
Hb at diagnosis (g/dL)	9.1	7.4	^1^ 6; ^2^ 9.2
RBC transfusions (n°)	3	2	2
RBC transfusion after first dose of rpFVIII (n°)	0	0	0
Increase in inhibitor rpFVIII titer	No	No	Yes
Thromboembolic events	None	None	None
Hb at discharge (g/dL)	9.3	10.5	^1^ 8.9; ^2^ 10.5
Bleeding relapse	No	No	No

Hb: hemoglobin; RBC: red blood cells; rpFVIII: recombinant porcine FVIII. (^1^ First hospitalization; ^2^ second hospitalization).

**Table 4:** Characteristic of Obizur^®^ treatment in periprocedural cases.

	Case 1	Case 2	Zanon et al. [25]	Buczuma et al. [28]
Weight (kg)	57.5	50	80	69
Total dose (U)	125.000	5000	136.000	108.000
Total exposures (days)	11	1	15	8
Total dose for weight (U/kg)	2174	100	1700	1565
Total dose for day (U/die)	11,364	5000	9067	13,500
Total dose for weight for day (U/kg/die)	198	100	113	196
FVIII level at diagnosis (%)	0.9	1.7	<1	N/A
hFVIII inhibitors titer (UB/mL)	76	23	110	0.64
Recovery FVIII level within 1 h (%)	125	94	51	173

**Table 5:** Characteristics of Obizur^®^ treatment in outpatient settings.

	Case 3	Tarantino et al. [18]	Tarantino et al. [18]
Weight (kg)	55	72	97
Total dose (U)	55.000	149.760	63.064
Total exposures (days)	22	26	14
Total exposures as outpatient (days)	16	16	6
Total dose for weight (U/kg)	1000	2080	650
Total dose for day (U/die)	2500	5760	4505
Total dose for weight for day (U/kg/die)	45	80	46
FVIII level at diagnosis (%)	1	<1	<1
hFVIII inhibitors titer (UB/mL)	64	32	N/A

## Data Availability

No new data were created or analyzed in this study. Data sharing is not applicable to this article.
